# A single-arm retrospective study of the clinical efficacy of unilateral biportal endoscopic transforaminal lumbar interbody fusion for lumbar spinal stenosis

**DOI:** 10.3389/fsurg.2022.1062451

**Published:** 2023-01-23

**Authors:** Xiangbin Wang, Zheng Tian, Maiwulan Mansuerjiang, Aikebaier Younusi, Leilei Xu, Haibin Xiang, Li Cao, Chong Wang

**Affiliations:** Department of Orthopaedics, The First Affiliated Hospital of Xinjiang Medical University, Urumqi, China

**Keywords:** lumbar spinal stenosis, unilateral biportal endoscopy technique, lumbar interbody fusion, spinal endoscopic surgery, minimally invasive

## Abstract

**Objective:**

The purpose of this study was to investigate the clinical efficacy of unilateral biportal endoscopic transforaminal lumbar interbody fusion (UBE-TLIF) for lumbar spinal stenosis (LSS).

**Methods:**

Patients who underwent UBE-TLIF due to single-segment LSS between August 2019 and July 2021 were retrospectively included in the study. Clinical outcomes evaluated include operative time, estimated blood loss (including postoperative drainage), time to ambulation, postoperative hospital stay, complications, visual analog scale (VAS) scores of low back pain and leg pain, Japanese Orthopaedic Association (JOA) score, Oswestry disability index (ODI), and modified Macnab criteria. Interbody bony fusion at the index level was assessed using Bridwell grading criteria.

**Results:**

A total of 73 patients (29 males and 44 females) were enrolled in this study. All surgeries were successfully performed without intraoperative conversion to open surgery. Magnetic resonance imaging (MRI) revealed optimal direct neural decompression after UBE-TLIF. The mean operative time was 150.89 ± 15.58 min. The mean estimated blood loss was 126.03 ± 17.85 ml (postoperative drainage was 34.84 ± 8.31 ml). Time to ambulation was 2.0 ± 0.75 days after the procedure. Postoperatively, the mean hospital stay was 5.96 ± 1.38 days. VAS scores of low back pain and leg pain, JOA, and ODI were significantly improved postoperatively compared with those before the operation, and differences were statistically significant (*P < *0.05). Excellent and good outcomes were reported by 87.67% of patients according to the modified Macnab criteria at the final follow-up. A total of nine perioperative complications occurred, with an incidence of 12.33%. X-ray or computerized tomography (CT) 6 months after the procedure showed that 37 cases (50.68%) presented with segmental fusion, 30 cases (41.10%) showed incomplete fusion, and 6 cases (8.22%) showed no signs of fusion. However, bony fusion was achieved in all cases at the final follow-up.

**Conclusions:**

UBE-TLIF for LSS has the advantages of less surgical invasiveness and fast postoperative recovery.

## Introduction

Lumbar spinal stenosis (LSS) is a disease caused by the compression of the dural sac and nerve root due to various factors such as hypertrophy of the ligamentum flavum (LF), facet joint hypertrophy, disc herniation, and spondylolisthesis, resulting in low back pain, leg pain with or without numbness, intermittent claudication, and bladder and bowel dysfunction, in which intermittent neurogenic claudication is the main feature. Degenerative LSS affects most commonly the elderly (1, 2). Conservative treatment is preferred for symptomatic LSS, while surgery may be considered for patients with severe radicular pain and walking disability who have failed to respond to conservative treatments, which accounts for approximately 8%–11% of degenerative lumbar spinal diseases that require surgical procedures (2–4). Traditional surgical approaches include open laminotomy decompression, foraminotomy, discectomy, and fusion (5–7). Conventional open lumbar decompression has a long history and has the advantages of adequate decompression and clear visualization of neural structures, while surgical invasiveness and extensive stripping of paraspinal muscles and soft tissues may lead to a series of problems such as postoperative low back pain, spinal instability, and prolonged hospital stay and time to return to normal life after the operation (8, 9). To address many of these shortcomings, innovative and less demolishing surgical techniques are being developed and investigated.

Minimally invasive spine surgery has become increasingly popular in recent years. Unilateral biportal endoscopy (UBE) was proposed by Heo in 2017 to treat degenerative lumbar spinal diseases with less damage to the paraspinal muscles (10). Unilateral biportal endoscopic transforaminal lumbar interbody fusion (UBE-TLIF) based on this technique is a newly emerging minimally invasive fusion surgery, and some studies have reported excellent outcomes in the treatment of LSS (10–13). Despite its recent introduction, the use of UBE is growing, thus requiring more clinical research to carefully evaluate outcomes related to this innovative technique. Consequently, this study was conducted to evaluate the clinical efficacy of UBE-TLIF by retrospectively analyzing clinical and radiological outcomes in a cohort of patients affected by LSS.

## Materials and methods

This was a single-arm retrospective study. The study protocol was approved by the Ethics Committee of the First Affiliated Hospital of Xinjiang Medical University and performed according to the Declaration of Helsinki. A total of 73 patients (29 men and 44 women) diagnosed with LSS and treated with UBE-TLIF between August 2019 and July 2021 were included in the study. All patients were informed of all potential risks of the surgery and signed written consent before the procedure.

The inclusion criteria are as follows: (1) definite diagnosis of LSS (central stenosis, lateral recess stenosis, and foraminal stenosis) with or without segmental instability (anterior translation [>3 mm], and/or increasing segmental sagittal motion [>15˚]), with or without low-grade lumbar spondylolisthesis (grade ≤ 2) on flexion/extension radiographs, including degenerative spondylolisthesis and isthmic spondylolisthesis; (2) patients with neurogenic claudication, pain, and numbness in the lower limbs, with or without low back pain, who have failed for more than 6 months of conservative treatment; (3) UBE-TLIF surgery; and (4) postoperative follow-up time ≥12 months. The exclusion criteria are as follows: (1) previous posterior decompression at the index level; (2) other concomitant spinal diseases (e.g., spinal infections, spinal tumors, and spinal trauma); (3) high-grade (Meyerding grade 3 or 4) isthmic spondylolisthesis and degenerative spondylolisthesis; (4) LSS involving two or more segments; and (5) presence of surgical contraindications.

### Surgical methods

**Figure 1:**
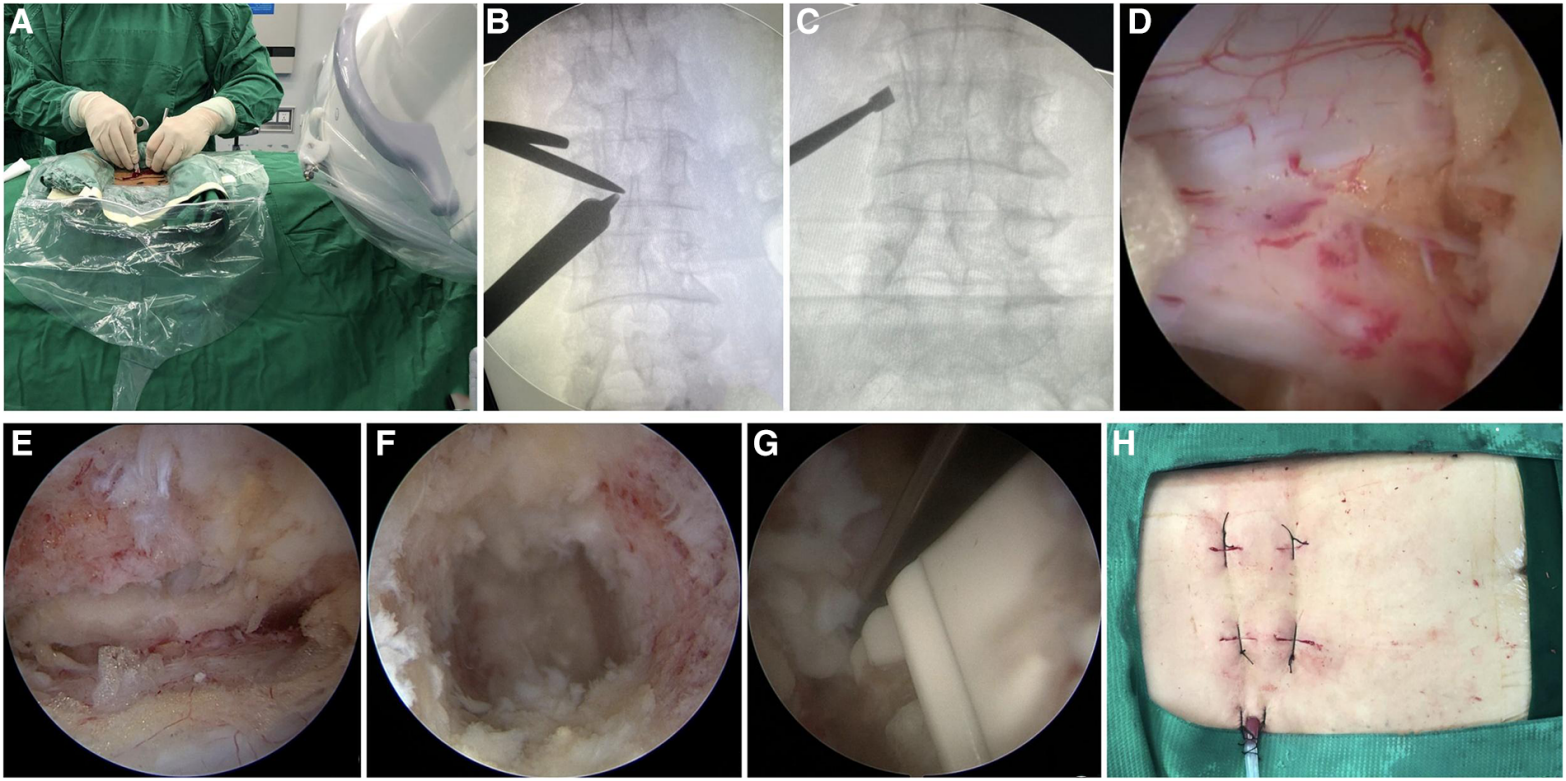
Intraoperative images of UBE-TLIF. (**A**) Operator creates two portals. (**B**) Location of the junction point of the serial dilators and the lamina dissector was confirmed by C-arm fluoroscopy. (**C**) Target segment was confirmed by C-arm fluoroscopy. (**D,E**) Endoscopic images of the dura, ipsilateral traversing root, and contralateral traversing root. (**F**) Endoscopic showed the intervertebral space with the cartilaginous endplate completely removed. (**G**) Cage was inserted under endoscope guidance. (**H**) Photo of the incision after completion of the operation.

All procedures were performed by the same surgical team. The patient was positioned prone on the operating table after achieving satisfactory general anesthesia. The target segment was identified, and portals were marked under C-arm fluoroscopy guidance, followed by skin asepsis and sterile draping. Two K-wires were inserted into the marked portals under fluoroscopy to confirm the disc space located at the target segment. Two longitudinal incisions of approximately 1.5 cm were made for viewing and working portals to introduce an arthroscope and surgical instruments, respectively. Two incisions were located 1 cm above and 1 cm below the center, where the two K-wires' junction points were located and placed close to the outer side of the pedicle. In left-sided approaches, the cranial portal was used as the viewing portal and the caudal portal was used as the working portal, while the opposite order was followed in right-sided approaches. Serial dilators and laminar dissectors were inserted through the portals and placed in direct contact with the bone, and the precise location was confirmed by fluoroscopy (Figures 1A,B). After soft tissue debridement with an arthroscopic shaver and careful hemostasis, an osteotome or a K-wire was inserted in the facet joint space or in contact with the bone surface, and the target segment location was again confirmed by fluoroscopy (Figure 1C). Ipsilateral laminectomy and facetectomy were performed first. Osteotomes, Kerrison punches, and high-speed burrs were used to remove the inferior articular process (IAP) and the inferior margin of the superior lamina to expose the origin of the LF, the superior margin of the inferior lamina to reveal the end of the LF, and then the apical and medial margins of the superior articular process (SAP). Subsequently, contralateral decompression was performed. Local autologous bone obtained during the procedure was saved for later use as an interbody bone graft. In case of insufficient autologous bone, artificial or allogenic bone grafts were used. The LF overlying the dura and nerve roots was removed following ipsilateral and contralateral decompression, and facetectomy was completed.

Subsequently, ipsilateral and contralateral nerve roots were explored to ensure adequate decompression (Figures 1D,E). Annulotomy was performed with a sharp knife following the dura and nerve root being protected and then discectomy with tools. The arthroscope was introduced into the intervertebral space to monitor the preparation of the endplate (Figure 1F), the cartilaginous endplate was removed completely with a curette, and the subchondral bone was exposed until the wound had blood ooze. A cage trial implant was inserted into the disc space to restore the intervertebral height while avoiding subchondral bone injury and to determine the size of the real cage. A special cannula was used to fill the anterior part of the disc space with local autogenous bone and artificial bone owing to the concern of bone loss caused by continuous irrigation. The cage was carefully inserted in the intervertebral disc space under arthroscopic observation to avoid injury to the dura and nerve roots (Figure 1G). Eventually, the adequateness of cage size and position was demonstrated by fluoroscopy. Subsequently, the arthroscope and endoscopic instruments were withdrawn, and ipsilateral pedicle screws were implanted via the viewing and working portals. Contralateral pedicle screws were placed percutaneously using conventional skin incisions. A surgical drain was positioned to drain small bony debris and prevent epidural hematoma, and incisions were sutured (Figure 1H).

### Postoperative management

**Figure 2:**
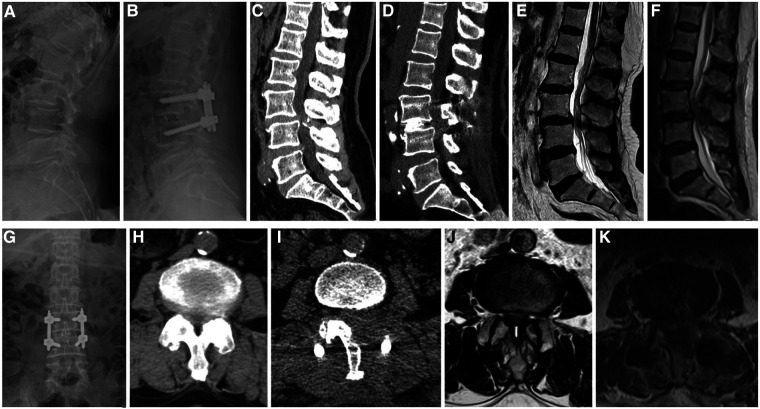
A 62-year-old female patient, whose complaints were low back pain since 3 years, lower limbs numbness, and intermittent claudication since 5 months. (**A,C,E**) Preoperative lateral radiographs, sagittal CT, and MRI showing instability of the L3 vertebral body, L3–4 spinal stenosis, and ossification of the posterior ligamentum flavum. (**H,J**) Preoperative axial CT and MRI showing significant spinal stenosis in L3–4. (**B,G**) Postoperative anteroposterior and lateral radiographs showing a good position of the instrumentation and the cage and improved segmental instability. (**D**) Postoperative sagittal CT showing that adequate bone was grafted. (**F**) Postoperative sagittal MRI showing that spinal stenosis was improved. (**I**) Postoperative axial CT showing unilateral laminectomy bilateral decompression. (**K**) Postoperative axial MRI showing sufficient decompression and a good position of the cage.

Intravenous antibiotic prophylaxis was administered for 24 h postoperatively, and nonsteroidal anti-inflammatory drugs (NSAIDs) were used to reduce pain. The drain tube was removed when the drain flow was <30 ml/24 h. The patients were allowed to walk with a brace 1 day postoperatively, and brace protection continued for 2–3 months. X-ray (Figures 2B,G) and computerized tomography (CT) (Figures 2D,I) were performed on all patients before discharge to evaluate the location of the graft and instrumentation, and adequateness and extent of decompression were assessed by sagittal and axial magnetic resonance imaging (MRI) (Figures 2F,K).

### Outcome measures

All patients were evaluated clinically and by x-ray, CT, and MRI (Figures 2A,C,E,H,J). Operative time, estimated blood loss (including postoperative drainage), time to ambulation, postoperative hospital stay, and complications were recorded and documented. Visual analog scale (VAS) scores of low back pain and leg pain, Japanese Orthopaedic Association (JOA) scores, and the values of Oswestry disability index (ODI) preoperatively and during the follow-up period (1 day, 1 month, 3 months, and 6 months after surgery, and the last follow-up) were recorded. Modified Macnab (14) criteria were appraised at the last follow-up. Intervertebral bony fusion was assessed using Bridwell grading criteria (15). When there was uncertainty in x-ray, further evaluation was done by CT.

### Statistical analysis

The data were statistically analyzed using SPSS 26.0 software. The continuous data were expressed as the mean ± standard deviation (SD), and significant differences in repeated-measures data (VAS, JOA, and ODI) were determined using repeated-measures analysis of variance. *P *< 0.05 was considered to be statistically significant.

## Results

A total of 73 patients (29 men and 44 women, 60.78 ± 7.29 years) that met the criteria were included in our study. All patients were followed for at least 12 months, and the average follow-up time was 17.92 ± 3.22 months. A total of 10 patients had central stenosis, 10 patients had central stenosis with lateral recess stenosis, 11 patients had central stenosis with concomitant foraminal stenosis, 16 patients had central stenosis with segmental instability, 16 patients had LSS with degenerative spondylolisthesis, and 10 patients had LSS with isthmic spondylolisthesis. The operative levels ranged from L2–3 to L5–S1: L2–3 in 7 patients, L3–4 in 15 patients, L4–5 in 32 patients, and L5–S1 in 19 patients (Table 1).

**Table 1:** Demographic and surgical characteristics of included patients.

Variables	Value
Age (years)	
Mean	60.78 ± 7.29
Range	45–75
**Gender**	
Male	29
Female	44
Follow-up times (months)	17.92 ± 3.22
**Diagnosis**	
Central stenosis with segmental instability	16
LSS with DS	16
Central stenosis with concomitant foraminal stenosis	11
Central stenosis with lateral recess stenosis	10
Central stenosis	10
LSS with IS	10
**Spondylolisthesis**	
DS	
Grade 1	13
Grade 2	3
** IS**	
Grade 1	6
Grade 2	4
**Level treated**	
L2–3	7
L3–4	15
L4–5	32
L5–S1	19
**Approach**	
Ipsilateral decompression	43
Bilateral decompression	30
**Approaching side**	
Left	45
Right	28

Values are presented as the number of patients unless stated otherwise.

DS, degenerative spondylolisthesis; IS, isthmic spondylolisthesis; LSS, lumbar spinal stenosis.

**Figure 3:**
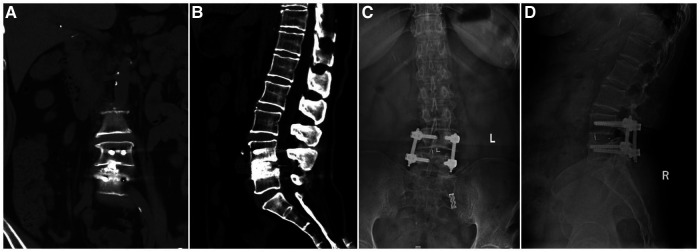
Imaging findings during follow-up of a patient who underwent UBE-TLIF. (**A,B**) Coronal and sagittal CT showing that the cage was well positioned and high-density bone fusion between vertebral bodies 6 months after the operation. (**C,D**) 13-month postoperative x-ray showing bony fusion and that the instrumentation was in a good position.

All patients completed the procedure successfully without intraoperative conversion to open surgery. The mean operative time was 150.89 ± 15.58 min. The mean estimated blood loss was 126.03 ± 17.85 ml (postoperative drainage was 34.84 ± 8.31 ml). The time to ambulation was 2.0 ± 0.75 days after the procedure. The mean postoperative hospital stay was 5.96 ± 1.38 days (Table 2). Preoperative VAS scores improved significantly after the surgery: the mean VAS scores of low back pain and leg pain were 5.23 ± 1.67 and 5.62 ± 2.25, respectively, before surgery, which improved to 3.03 ± 1.25 and 3.62 ± 1.90 the next day after surgery (*P* < 0.05). The VAS scores of low back pain and leg pain were 2.10 ± 1.23 and 2.58 ± 1.50, respectively, 1 month after the operation, which improved significantly over the corresponding preoperative values (*P* < 0.05). The VAS scores of low back pain and leg pain were 1.53 ± 0.96 and 1.52 ± 1.0, respectively, 3 months after the operation, which improved significantly over the corresponding preoperative values (*P* < 0.05). The VAS scores of low back pain and leg pain were 1.23 ± 0.94 and 1.01 ± 0.66, respectively, 6 months after the operation, which improved significantly over the corresponding preoperative values (*P *< 0.05). The final VAS scores of low back pain and leg pain were 0.96 ± 0.77 and 0.93 ± 0.75, respectively (*P *< 0.05). Postoperative JOA scores significantly improved compared to preoperative scores: the mean JOA score was 10.75 ± 2.23. The 1-month JOA score was 19.30 ± 2.18 (*P* < 0.05). The 3-month JOA score was 21.07 ± 1.80 (*P* < 0.05). The 6-month JOA score was 23.12 ± 1.76 (*P* < 0.05). The final JOA score was 27.01 ± 1.31 (*P* < 0.05). Moreover, the preoperative ODI score (65.73 ± 8.29) also improved significantly at the follow-up (*P *< 0.05). The 1-month ODI score was 45.66 ± 8.22 (*P* < 0.05). The 3-month ODI score was 35.76 ± 7.93 (*P* < 0.05). The 6-month ODI score was 22.81 ± 3.60 (*P* < 0.05). The final ODI score was 9.67 ± 2.42 (*P* < 0.05) (Table 3). Based on the modified Macnab criteria at the final follow-up, the clinical outcomes were excellent in 50 (68.49%) patients, 14 (19.18%) patients had good clinical outcomes, 9 (12.33%) patients had fair clinical outcomes, and none of the patients showed poor outcomes. In total, 87.67% showed excellent to good outcomes, and 12.33% showed fair outcomes (Table 4). X-ray or computerized tomography (CT) (Figures 3A,B) 6 months after the procedure showed that 37 cases (50.68%) presented with segmental fusion, 30 cases (41.10%) showed incomplete fusion, and 6 cases (8.22%) showed no signs of fusion. However, bony fusion was achieved in all cases at the final follow-up (Figures 3C,D). No loosening or fracture of the internal fixation occurred in all patients.

**Table 2:** Results related to UBE-TLIF.

Variable	Value
Operative time (min)	150.89 ± 15.58
Estimated blood loss (ml)	126.03 ± 17.85
Postoperative drainage (ml)	34.84 ± 8.31
Time to ambulation (days)	2.0 ± 0.75
Postoperative hospitalization time (days)	5.96 ± 1.38

**Table 3:** Clinical outcomes (VAS, JOA, and ODI) pre- and post-surgery.

Date	VAS score of low back pain	VAS score of leg pain	JOA score	ODI score (%)
Preoperative	5.23 ± 1.67	5.62 ± 2.25	10.75 ± 2.23	65.73 ± 8.29
**Postoperative**				
1 day	3.03 ± 1.25^a^	3.62 ± 1.90^a^	-	-
1 month	2.10 ± 1.23^a^	2.58 ± 1.50^a^	19.30 ± 2.18^a^	45.66 ± 8.22^a^
3 months	1.53 ± 0.96^a^	1.52 ± 1.0^a^	21.07 ± 1.80^a^	35.76 ± 7.93^a^
6 months	1.23 ± 0.94^a^	1.01 ± 0.66^a^	23.12 ± 1.76^a^	22.81 ± 3.60^a^
Final follow-up	0.96 ± 0.77^a^	0.93 ± 0.75^a^	27.01 ± 1.31^a^	9.67 ± 2.42^a^
*p*-Value	*P* < 0.05	*P* < 0.05	*P* < 0.05	*P* < 0.05

VAS, visual analog scale; JOA, Japanese Orthopaedic Association; ODI, Oswestry disability index.

^a^ Significantly different from the preoperative value (*P* < 0.05).

**Table 4:** Clinical outcome of surgery based on modified Macnab criteria.

Classification	Frequency (%)
Excellent	50 (68.49)
Good	14 (19.18)
Fair	9 (12.33)
Poor	–

We observed nine cases of perioperative complications: three patients with postoperative epidural hematoma, two patients with a dural tear, two patients with transient pain in the buttocks, one patient with temporary dysesthesia, and one patient with transient muscle paralysis of both lower limbs, in which the incidence of complications was 12.33% (Table 5). None of these patients underwent revision surgery, and their complications recovered after conservative treatment. No infection was observed in our patients.

**Table 5:** Complications of included patients.

Complication	Value	Incidence (%)
Postoperative epidural hematoma	3	4.11
Dural tear	2	2.74
Transient pain in the buttocks	2	2.74
Temporary dysesthesia	1	1.37
Transient muscle paralysis of both lower limbs	1	1.37
Total	9	12.33

## Discussion

LSS is a common degenerative lumbar spinal disease in the elderly, whose incidence rate is accruing every year, and patients' expectations from surgery are also improving. Although traditional open transforaminal lumbar interbody fusion (TLIF) and posterior lumbar interbody fusion (PLIF) can be effective treatments for LSS by directly decompressing the spinal canal through the posterior approach, disruption of the posterior muscles and ligamentous structures may lead to complications such as postoperative low back pain and muscle atrophy (16, 17). Therefore, more time may be required for functional recovery after conventional open fusion surgery, resulting in relatively longer postoperative hospital stays and higher costs associated with postoperative care. Consequently, minimally invasive fusion techniques such as oblique lumbar interbody fusion, percutaneous endoscopic lumbar interbody fusion, and minimally invasive transforaminal lumbar interbody fusion (MI-TLIF) have been developed to minimize the procedure-related injuries of posterior muscles and ligamentous structures (16, 18–20).

The UBE technique has been recently introduced with different applications, including decompression and interbody fusion (11, 21–29). It is based on using two independent portals (viewing and working) requiring two small incisions. Lately, UBE to perform TLIF (here defined as UBE-TLIF) has been described (10, 11). This technique has some advantages such as a clear view, wide working space, and operative freedom, additionally allowing the use of conventional spinal surgical tools for decompression, which combines the features of endoscopic surgery with those of traditional open surgery and truly embodies the minimally invasive concept. It does not require a tubular retractor during the procedure, similar to traditional open spine surgery, and the extent of intraoperative decompression can be evaluated as needed. It is less disruptive to normal bony structures than conventional open TLIF and therefore provides a reduced quantity of local autologous bone, which is usually insufficient to achieve strong intervertebral fusion. However, according to the authors' experience, an adequate amount of bone graft can be obtained during decompression by sequentially removing the IAP, the lower edge of the superior lamina, the upper edge of the inferior lamina, as well as the apical and medial of the SAP. After determining the approximate position of the pedicle with a probe hook during resection of the SAP, an osteotomy can be performed with an oscillating saw or an ultrasonic osteotome. This allows to both reduce cancellous bone bleeding and also obtaining a decent quantity of bone graft, avoiding the loss of small bone fragments caused by continuous flush. Secondly, minimizing the frequency of using burr during the procedure will consent to save a larger amount of bone graft. In addition, a synthetic or allogenic bone graft may be used in case of insufficient autologous bone. When contralateral decompression is performed, we recommend removing first the inferior aspect of the spinous process with an osteotome or high-speed burr using a protective sheath to reduce the risk of dural damage. A curette or Kerrison rongeur may be helpful to remove the contralateral LF. Crossing the midline of the spinous process to reach the contralateral lateral recess, probing the medial wall of the contralateral pedicle, and ensuring that the dural sac and nerve roots are free to move to indicate that the decompression is complete. Preserving the LF is undoubtedly safer; however, in cases where only ipsilateral decompression is required, flavectomy at an early stage provides a wider operative view and helps avoid disorientation during the procedure. However, when performing contralateral decompression, we recommend temporary preservation of ipsilateral LF to reduce the risk of dural and ipsilateral nerve root injury. In particular, in cases with severe LSS, if the ipsilateral LF is removed first, significant expansion of the dural sac can lead to “overtopping” difficulty and increase the risk of injury.

There is a lack of multicenter, large-sample, prospective studies on the efficacy of UBE-TLIF in treating LSS. The concept of the UBE technique was introduced and used for lumbar interbody fusion by Heo (10) in 2017. A total of 69 patients who underwent single-level fusion were reported with an average age was 71.2 years, estimated blood loss was 85.50 ± 19.40 ml, operative time was 165.80 ± 25.50 min, and the follow-up period was 13.5 months. Postoperative MRI showed optimal direct neural decompression, the VAS score and ODI significantly improved, and no case of neurological deterioration was encountered. Kim (11) adopted UBE-TLIF for 14 cases in 2018. The average age of these patients was 68.7 years, postoperative blood loss was 74.0 ± 9.0 ml, operative time was 169.0 ± 10.0 min, and the preoperative VAS score was 7.40, which decreased to 2.70 at 2 months postoperatively. In 2019, Park (25) compared the 1-year follow-up efficacy of UBE-TLIF and conventional PLIF for degenerative lumbar spinal diseases. The mean operative time of the UBE-TLIF group (158.0 min) was longer than that of the PLIF group (137.0 min), and there were significantly more transfusion cases in the PLIF group (20%) than in the UBE-TLIF group (no case). There was a significant improvement in the VAS score of low back pain in the UBE-TLIF group at 1 week, which was significantly better than the PLIF group, but the VAS score of low back pain among patients preoperatively and 1 year postoperatively did not show a statistically significant difference. The VAS scores of leg pain and ODI significantly improved postoperatively in both groups. The clinical results of UBE-TLIF and MI-TLIF in patients with single- or two-segment LSS with or without lumbar spondylolisthesis were compared by Kang (26) in 2021. The VAS score of low back pain and the SF-36 score were more significantly improved in the UBE-TLIF group than the MI-TLIF group at 1 month postoperatively. Nevertheless, the mean VAS scores of low back pain and leg pain, the ODI, and the SF-36 score were not significantly different between groups 1 year after the procedure. Although the total operative time was significantly longer in the UBE-TLIF group, the estimated blood loss and the amount of surgical drainage were significantly more in the MI-TLIF group.

A total of 73 patients completed the procedure in our study. UBE-TLIF is superior to conventional open lumbar fusion reported in an article in terms of estimated blood loss, time to ambulation, and postoperative hospital stay (25). UBE-TLIF operative time is longer than conventional open surgery but shorter than MI-TLIF, as reported by Kim et al. (13), and is probably due to the steep learning curve. Surgeons need to become familiar with the endoscopic anatomy of the spine and carefully stop bleeding to maintain a clear surgical field during the operation. Moreover, discectomy and endplate preparation are often time-consuming surgical steps, especially during early cases (30). A study reported that the technique requires approximately 34 cases to reach an appropriate level of stability (13).

Biportal endoscopic decompression for LSS of 104 and 58 cases was reported by Soliman (21) and Hwa (3) in 2015 and 2016, respectively. UBE has been increasingly used to treat degenerative lumbar spine diseases with wider applications and more satisfactory outcomes. The rate of serious complications associated with the procedure also decreased significantly as the techniques matured. A dural tear is one of the most common complications during endoscopic decompression, with a reported incidence of up to 13.20% (31), while in our study, only two cases (2.74%) of dural tears were encountered. In both cases, the tears were repaired with a gelatin sponge, the skin incision was tightly sutured, and the compressive dressing was applied. In one case, the dural tear occurred during the removal of a central calcified herniated nucleus pulposus and involved the ventral aspect of the dural sac from ipsilateral to contralateral. In the other case, a small dural defect developed during contralateral decompression while removing the LF from the inferior lamina with a Kerrison rongeur. Three patients with a low volume of postoperative drain had a recurrence of leg pain shortly after the drain tube was removed, which occurred because of epidural hematoma formation. However, symptoms completely disappeared after conservative treatment. Two patients who had undergone unilateral laminectomy and bilateral decompression had mild buttock pain the day postoperatively, while this was not reported preoperatively. We hypothesize that symptoms may have been caused by cauda equina stimulation due to the “overtopping” process during contralateral decompression. Nonetheless, symptoms spontaneously resolved after observation. One case presented with temporary dysesthesia in the anterolateral aspect of the left leg and dorsum of the foot with no movement impairment. Also, in this case, symptoms spontaneously resolved after observation. One patient had transient muscle paralysis in both lower limbs as a result of significant intraoperative strain on the dural sac and nerve roots due to the inappropriate retraction at the beginning of the learning curve. Dehydrating drugs, neurotrophic drugs, and functional exercise of lower limbs were used after the operation. Muscle strength was partially improved after 1 week and returned to normal 1 month postoperatively.

A study concluded that the complication rate of UBE decompression of LSS was 6.3% (32). Pranata et al. (33) summarized that the complication rates of UBE and microscopic decompression for LSS were comparable. In another research, Park compared the clinical and radiological outcomes of UBE-TLIF and conventional PLIF for degenerative lumbar spine disease, which summarized that UBE-TLIF was less invasive than PLIF but as effective as conventional PLIF in improving clinical outcomes and obtaining fusion (25). These studies reaffirm the safety and effectiveness of the UBE technique in the treatment of LSS, and it has an extensive surgical view and sufficient operative space to enable traditional open decompression surgery to be performed endoscopically. Combined with the above-mentioned effectiveness, safety, and several advantages, the authors deem that the UBE technique has broad prospects. Nevertheless, the conclusions of this study need to be further validated by the accumulation of more cases and multicenter follow-up results due to this study being a retrospective study with a small sample size and a lack of multicenter studies. The results of this study showed a high complication rate at the beginning of the learning curve and a lack of comparative studies with other fusion procedures to demonstrate the effectiveness and safety of this technique. Furthermore, this study requires further validation of its long-term efficacy and radiological outcomes, including the long-term effects on spinal stability.

## Conclusion

UBE-TLIF for LSS has the advantages of less surgical invasiveness and faster postoperative recovery, which is an effective and safe minimally invasive fusion procedure that can provide a reference for treatment options for LSS.

## Data Availability

The original contributions presented in the study are included in the article/Supplementary Material; further inquiries can be directed to the corresponding author/s.
